# Mysterious seismoacoustic signals of eastern Helwan quarry blasts 2022

**DOI:** 10.1038/s41598-025-98694-6

**Published:** 2025-05-27

**Authors:** Islam Hamama, Shimaa H. Elkhouly, Hanan Gaber, Mona Hamada, Hany S. Elbehiri, Adel S. Othman, Mohamed Maklad, Mona Abdelazim, Mohamed N. ElGabry, Ahmed Lethy, Hesham Hussein, Masa-yuki Yamamoto

**Affiliations:** 1 Egyptian National Data Center, National Research Institute of Astronomy and Geophysics, Helwan, Cairo 11421 Egypt; 2 School of Systems Engineering, Kochi University of Technology, Kami, Kochi 782-8502 Japan; 3 Department of Physics and Astronomy “Augusto Righi” (DIFA), Alma Mater Studiorum - University of Bologna, Bologna, Italy

**Keywords:** Geophysics, Seismology

## Abstract

Mysterious seismoacoustic events were reported at the beginning of 2022 near Helwan Cairo, Egypt. The majority of these events were recorded by the Egyptian National Seismic Network. The source characteristics of the events were unknown. In May 2022, a temporary infrasound array station was established with a small aperture of 450 m in Helwan. Throughout the 6-month monitoring period, we employed a recursive short-term average/long-term average trigger method across all sensors, leading to the detection of the impulsive seismoacoustic events. Infrasound propagation models, coupled with F-K analysis, further confirmed the locations and directions of the recorded events, providing robust data that could be correlated with planet satellite images of the azimuth directions detected via the infrasound array analyses. The mysterious signals were identified as originating from a major construction project in Egypt: the high-speed railway train corridor. Our study demonstrates the effectiveness of integrating seismic sensors with infrasound arrays for enhanced source characterisation. The combination of these tools enabled precise discrimination of quarry blasts in eastern Helwan. Additionally, our findings suggest that inexpensive sensors can be a cost-effective solution for monitoring higher-frequency events.

## Introduction

Seismoacoustic detections have been reported from several experimental explosions. The Sayarim Infrasound Calibration experiments in 2009 and 2011 involved calibrating charges ranging from 7.4 to 96 tons of TNT. Additionally, various chemical explosions with yields between 90 kg and 45 tons of TNT equivalent (TTE) were conducted during the forensics surface experiment and the humming roadrunner experiment^1,2^. Furthermore, studies have analysed the Democratic People’s Republic of Korea explosion in 2013 and the Buerit-Port explosion in 2020. Such significant explosions produce acoustic signals that can travel long distances. The detectability of these signals is influenced by atmospheric conditions and wind noise levels around micro-barometer sensors^3,4^. The international monitoring system of the comprehensive nuclear test ban treaty includes 60 infrasound stations capable of detecting explosions with a yield of 1 kiloton TNT equivalent (KTE) at a minimum of two stations^5^. However, at a local level, quarry blasts are typically monitored by seismic networks. During these surface explosions, a portion of the energy is released into the atmosphere, creating a noticeable acoustic signature. Distinguishing between these blasts and natural earthquakes remains a complex task. While various seismic discrimination techniques have been documented, none of them offer direct seismic discrimination^6^. On the other hand, infrasound signals generated by earthquakes can be categorised as local or diffracted. The coupling of local infrasound from earthquakes depends on factors such as magnitude, depth, and the source mechanism. In the case of oceanic earthquakes, the presence of a T-phase at hydro-acoustic stations increases the likelihood of seismic signals coupling to acoustics in the atmosphere^7–10^. Given that acoustic signals are continuously generated from quarry blasts, infrasound technology can serve as a valuable tool for differentiating artificial events from natural earthquakes. In Egypt, investigations have classified mining quarry zones to better understand the nature and characteristics of seismic signals. The majority of these quarries are located in the northern part of the country^11^. Furthermore, there are active blasts in the southern region, near Aswan^12^. The National Research Institute of Astronomy and Geophysics monitors cement quarries around the greater Cairo area to ensure the safety of infrastructure and buildings, particularly for large-scale projects such as the new administrative capital^13^. In this study, we focus on the use of infrasound as a remote sensing tool to investigate a number of mysterious impulsive events near Helwan between 12 May and 31 December 2022. The majority of these events were recorded by the Egyptian National Seismic Network (ENSN)^14^ catalogue. A temporary infrasound array station was deployed in Helwan (coordinates: 29.86N, 31.33E). A short-time-average over long-time-average (STA/LTA) method was applied to continuous infrasound data to identify these events. In addition, frequency-wavenumber (F-K) analysis was done to estimate the azimuth directions of the signals. Moreover, satellite images were used to verify the effects of these events. Finally, infrasound propagation models were calculated from the study area toward the Helwan infrasound array to confirm the results. The mysterious seismoacoustic events were generated from quarry blasts near $\sim$ 20 km from the array, related to the construction of the high-speed train railway near Helwan.

## Helwan infrasound array

The Helwan array consists of five sensors (HLW, HL1, 668S, 669S, and 664S) with 450 m apertures arranged in a triangular configuration. Different types of infrasound sensor are utilised within the array. There are two adjacent MB3d sensors, one of which is integrated into the main element and equipped with a noise-reduction system consisting of 96 stainless steel pipes arranged into a Rosetta shape. The other does not have the noise-reduction system. The remaining three sensors are from Kochi University of Technology. The INF04 consists of an array of condenser microphones and was originally developed for deployment on the MOMO sounding rocket in Japan to study the upper atmosphere. A Raspberry Pi is used as a data logger. This system was used to detect the shockwave of the re-entry of the Hayabusa2 capsule in 2020^15,16^ and the OSIRIS-REx capsule in 2023^17^.

**Fig. 1:**
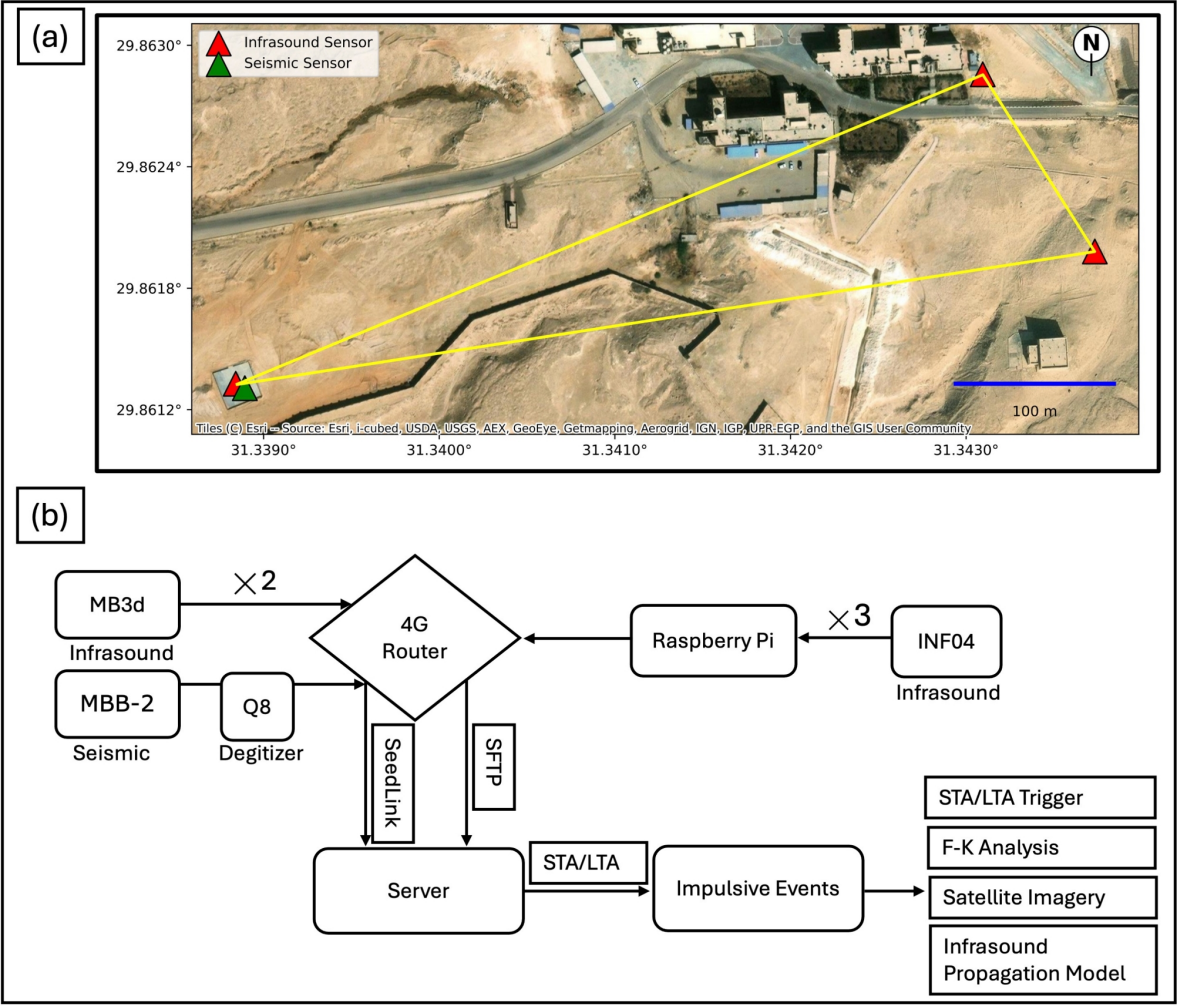
(**a**) Setup of the Helwan array and distribution of infrasound and seismic sensors (map plotted using the contextily python library^21^). (**b**) Flowchart of data analyses.

**Fig. 2:**
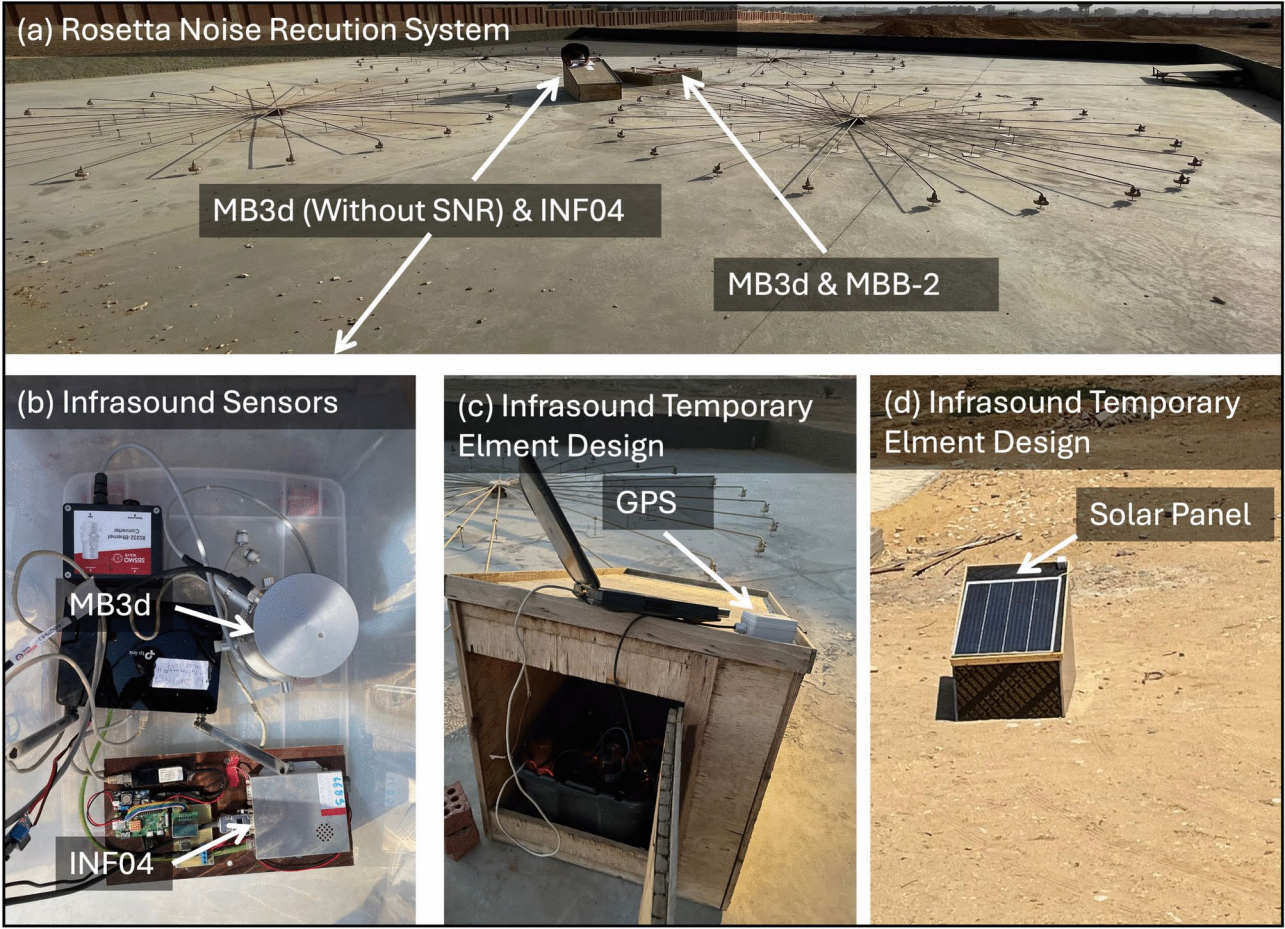
(**a**) Deployment of a noise-reduction system with an MB3d sensor near the HLW element. (**b**) Establishment of the MB3d (HL1) and INF04 (668S) infrasound sensors. (**c**, **d**) Design of the temporary infrasound sensors.

The data loggers continuously stream data to our centre via the Transmission Control Protocol using seed-link and secure file transfer protocols through 4G modems. An MBB-2 seismometer (HLW1) was added to the array as a collocated sensor with the MB3d main sensor in November 2022. Table 1 shows the location and station codes for the sensors of the Helwan array. The observation campaign using these seismoacoustic sensors has helped clarify the nature of impulsive signals. Figures 1 and 2 illustrate the infrasound elements of the Helwan array. The dataflow of our acquisition and processing operational phases is depicted in Fig. 1b.

**Table 1:** Sensor codes and coordinates of Helwan array elements.

Sensor code	Type	Latitude	Longitude	Technology
HLW	MB3d	29$^{\circ }$ 51 40.9 N	31$^{\circ }$ 20 20.0 E	Infrasound
HLW1	MBB-2	29$^{\circ }$ 51 40.9 N	31$^{\circ }$ 20 20.0 E	Seismic
668S	INF04	29$^{\circ }$ 51 40.9 N	31$^{\circ }$ 20 20.0 E	Infrasound
HL1	MB3d	29$^{\circ }$ 51 40.9 N	31$^{\circ }$ 20 20.0 E	Infrasound
669S	INF04	29$^{\circ }$ 51 46.3 N	31$^{\circ }$ 20 39.3 E	Infrasound
664S	INF04	29$^{\circ }$ 51 43.9 N	31$^{\circ }$ 20 39.6 E	Infrasound

## Methods

### Trigger method

The study focused on continuous data recorded from mid-May to the end of December 2022. The STA/LTA trigger method was applied to identify the impulsive signals within this period. This method is commonly used for weak-motion earthquakes and is efficient at sites with low noise levels. The underlying principle of STA/LTA is the continuous comparison of the amplitude of the noise level. The ratio of the STA/LTA trigger and detrigger thresholds can govern the recognition of the event waveform^18^. Recursive STA/LTA improves on the classical STA/LTA algorithm. The memory consumption during calculations is significantly reduced with this method, as it produces an exponentially decaying impulse response rather than a rectangular one. Furthermore, this method restricts shadow zones^19,20^. Thus, we applied recursive STA/LTA to the Helwan dataset, setting the lengths of the short- and long-term average windows to 2.5 and 10 s, respectively. Furthermore, we adjusted the threshold values for trigger and detrigger between 3.5 and 2.5. After obtaining the trigger times at all infrasound sensors, we declared an event when the difference in trigger time between each pair of sensors was < 2 s, and at least three sensors verified this condition. The resulting detections were then compared against the ENSN quarry bulletin of 2022.

### Power spectral density

The Welch method provides a robust estimation of the power distribution of five infrasound sensors across the data frequencies^22^. Therefore, we applied this method using Scientific Python^23^ to calculate power spectral densities (PSDs) with 128 samples of overlap. The method uses the fast Fourier transform algorithm to estimate the power spectra, which divides the data into fixed segments with a length of 512 samples. Moreover, each segment is converted from the time domain into the frequency domain using the algorithm. The average of the modified periodograms of these sections was evaluated.

### Frequency-wavenumber (F-K) analysis

F-K analysis is a commonly used technique in seismic array studies. The data are processed in the F-K domain, and the analysis identifies the direction of wave propagation. In addition, it can differentiate between recorded phases according to their propagation speeds. A Fisher detector was applied to estimate the direction of infrasound signals from lightning^24^. However, while this technique is useful for identifying the characteristics of wave propagation, there are some difficulties that can affect the detections, including array geometry, the weighting of each sensor in the calculations, and the spectral smoothing method employed^25^. In this study, the array processing function of the ObsPy package^26^ was used. The following equations (1–8) describe the array processing function and the steps involved in the calculations^27^. Assuming that infrasound propagates as a planar wave, the first step is conducting Fourier transform to convert the data into a frequency domain. The spatial and temporal spectrum $S(k_x, k_y, f)$ can be estimated from:

1 $$\begin{aligned} S(k_x, k_y, f) = \sum _{i=1}^{N} s_i(t) e^{-j (k_x x_i + k_y y_i)} e^{-j 2 \pi f t} \end{aligned}$$

where $k_x, k_y$ are the spatial wavenumbers in the $x$- and $y$- directions. $x_i, y_i$ are the positions of the $i$-th and $j$-th sensors in the array, $s_i(t)$ is the time-domain signal from the $i$-th sensor, and $f$ is the frequency. The steering vector $\mathbf{v}(\theta )$ in the direction $\theta$ is used in beamforming to obtain the model response of the array for waves arriving from different directions:

2 $$\begin{aligned} \mathbf{v}(\theta ) = \left[ e^{j k_1 \cdot \mathbf{r}_1}, e^{j k_2 \cdot \mathbf{r}_2}, \dots , e^{j k_N \cdot \mathbf{r}_N} \right] ^T \end{aligned}$$

where $\mathbf{r}_i$ are the position vectors of the sensors in the array, $N$ is the number of arrayed sensors, and $k_i$ is the wave number. The covariance matrix $R_{ij}(f)$ shows the relationship between the signals from different sensors in the array, which can be estimated as:

3 $$\begin{aligned} R_{ij}(f) = \langle F_i(f) \cdot F_j^*(f) \rangle \end{aligned}$$

where $F_i(f)$ is the Fourier transform of the $i$ signal, and $F_j^*(f)$ is the complex conjugate of the Fourier transform of the $j$ sensor. In addition, Capon beamforming was used to estimate the control $R_{ij}(f)$ and minimise the variance of the output, as follows:

4 $$\begin{aligned} P_{ij}(\theta , f) = \frac{1}{\mathbf{e}^H R_{ij}(f)^{-1} \mathbf{e}} \end{aligned}$$

$P_{ij}(\theta , f)$ is the beamforming power at frequency $f$ and direction $\theta$, $\mathbf{e}$ is the steering vector corresponding to the direction $\theta$, and $R_{ij}(f)$ is the covariance matrix at frequency $f$. $R_{ij}(f)^{-1}$ is the inverse of the covariance matrix. The slowness and back-azimuth can be determined from the following:

5 $$\begin{aligned} & \text {slow}_x = sll_x + ix \cdot sl_s \end{aligned}$$

6 $$\begin{aligned} & \text {slow}_y = sll_y + iy \cdot sl_s \end{aligned}$$

where $\text {slow}_x$ and $\text {slow}_y$ are the slowness in the $x$- and $y$- directions at grid points $ix$ and $iy$. $sll_x$ and $sll_y$ are the starting slowness values in the $x$- and $y$- directions. $sl_s$ is the slowness step.

7 $$\begin{aligned} & \text {slow} = \sqrt{{\text {slow}_x}^2 + {\text {slow}_y}^2} \end{aligned}$$

8 $$\begin{aligned} & \text {baz} = 180 \cdot \frac{tan^{-1}(\text {slow}_x, \text {slow}_y)}{\pi } \end{aligned}$$

where $\text {baz}$ (deg) is the back-azimuth, representing the direction of arrival of the infrasound wave relative to the north direction.

### Infrasound propagation modelling

Numerical modelling is used to investigate the infrasound propagation paths in the atmosphere. The parabolic equation (PE) has been used in atmospheric acoustics since the 1980s. The derivation of the PE solver was discussed by Waxler and Assink^28^ for range-dependent problems. The ePape module of the NCPAprop package includes the effect of topographic features specifically for long-range propagation distances^29^. The transmission losses can be estimated from ePape and can be described as: $TL(r, z) = 20 \log _{10} \left( \frac{|p(r, z) |}{p_0(r_0, z_0)} \right)$, where r is the source-to-receiver along-ground distance, z is the receiver altitude, $r_{0}$ is a reference distance, and $z_{0}$ is a reference altitude.

In this study, 1D PE models of daily atmospheric profiles were estimated at 9:00 UTC in the direction toward Helwan array for the period between May to the end of December 2022 over the quarries.

## Results

The availability of the infrasound data from all sensors exceeded 90% during the study period. Daily data were received continuously at our centre in Helwan. Several methods were used to investigate these mysterious events.

### Triggered events

**Fig. 3:**
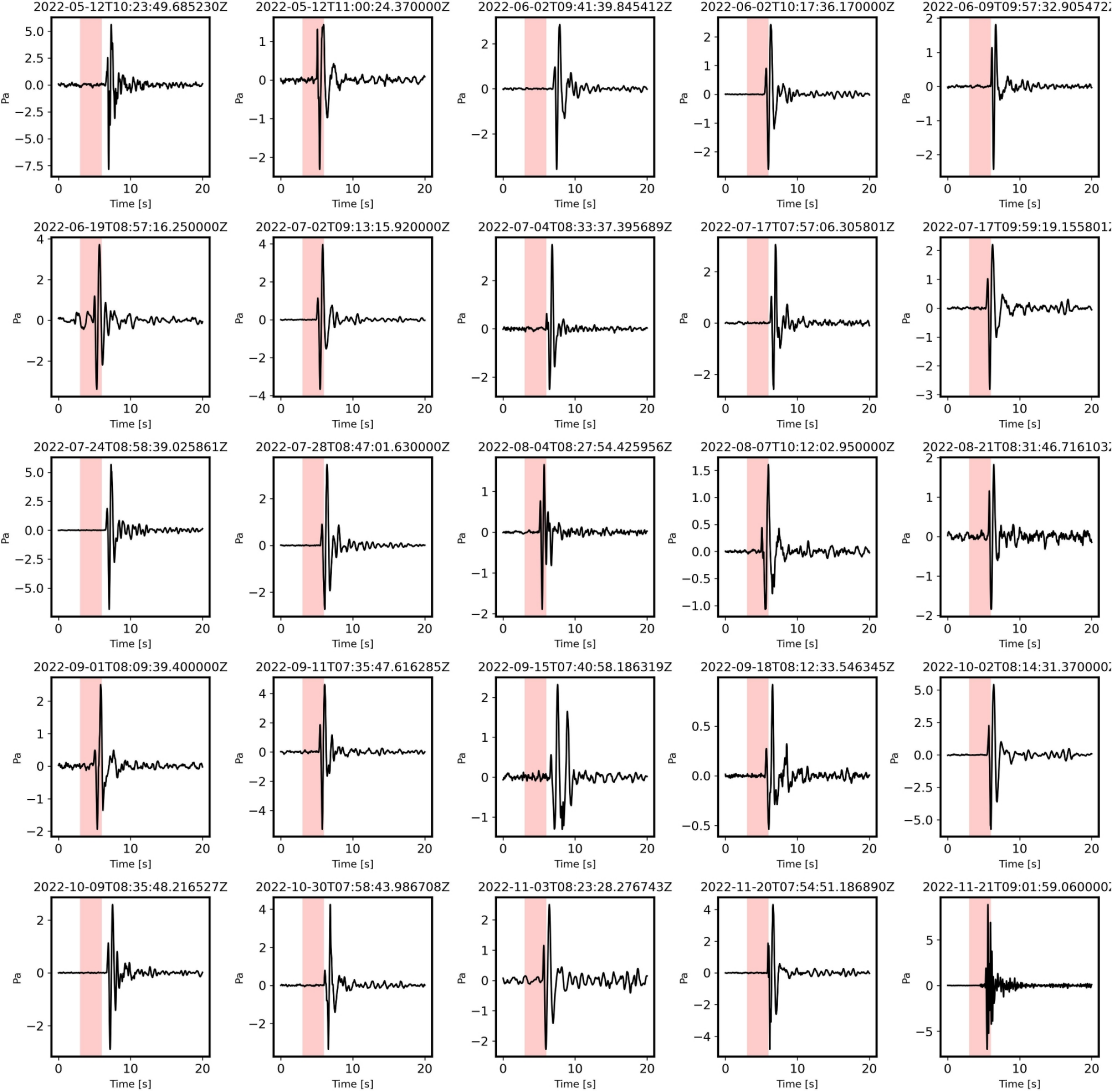
Examples of the impulsive signals detected after applying the STA/LTA recursive algorithm. The signals were filtered between 0.5 and 6 Hz.

The STA/LTA recursive algorithm was used to extract the impulsive signals from the continuous data. The trigger detector identified 6651 impulsive peaks from the infrasound sensors. After comparing arrival times from the five sensors, 101 events were identified as impulsive ones, having been detected by at least three sensors. Overall, 85 events matched the ENSN catalogue. In addition, 17 events only contained acoustic signals. The triggered peaks of the infrasound signals were systematically analyzed to verify the consistency of the time difference between the impulsive infrasound events and the seismic origin times listed in the quarry catalog. This time difference ranged from 25 to 34 s, accounting for minor shifts in the quarry locations as the high-speed railway corridor was being constructed, transitioning from the southeast to the northeast. Furthermore, the azimuthal directions were required to consistently correspond with the seismic quarry locations in Eastern Helwan. Figure 3 shows some examples of the detected events at the HLW sensor.

**Fig. 4:**
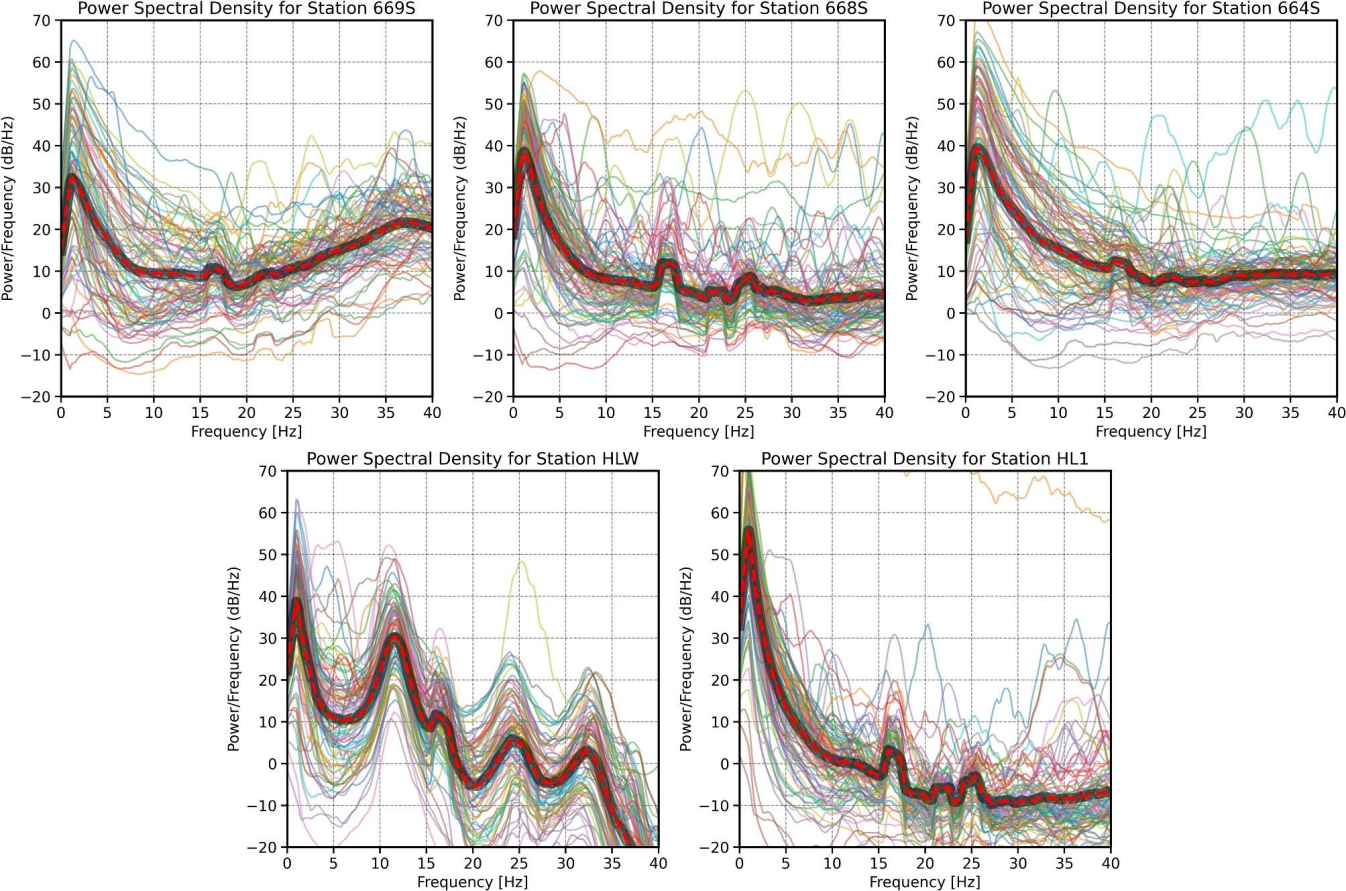
Comparison of the average PSDs across the five infrasound sensors for a 10 min duration centred around each triggered event.

We estimated PSDs for all triggered events using a 10-min duration waveform centred around each triggered arrival. Figure 4 shows the PSDs for infrasound sensors 669S, 668S, 664S, HLW, and HL1. High coherence was observed in different frequency bands. Significant peaks were noted at frequency bands lower than 6 Hz with power amplitudes of up to 55 dB. These peaks may be attributable to the triggered impulsive signals. Other peaks at 17.5 Hz were also recognised with lower power amplitudes of $\sim$ 10 dB. Furthermore, within a frequency band of 25 Hz, the results for HL1, 668S, and HLW indicated weak power, which might have been caused by noise near these three sensors, as they are installed next to each other. The power at frequencies above 17.5 Hz at the HLW site is lower than that of the other sensors, as this sensor includes the noise-reduction system.

**Fig. 5:**
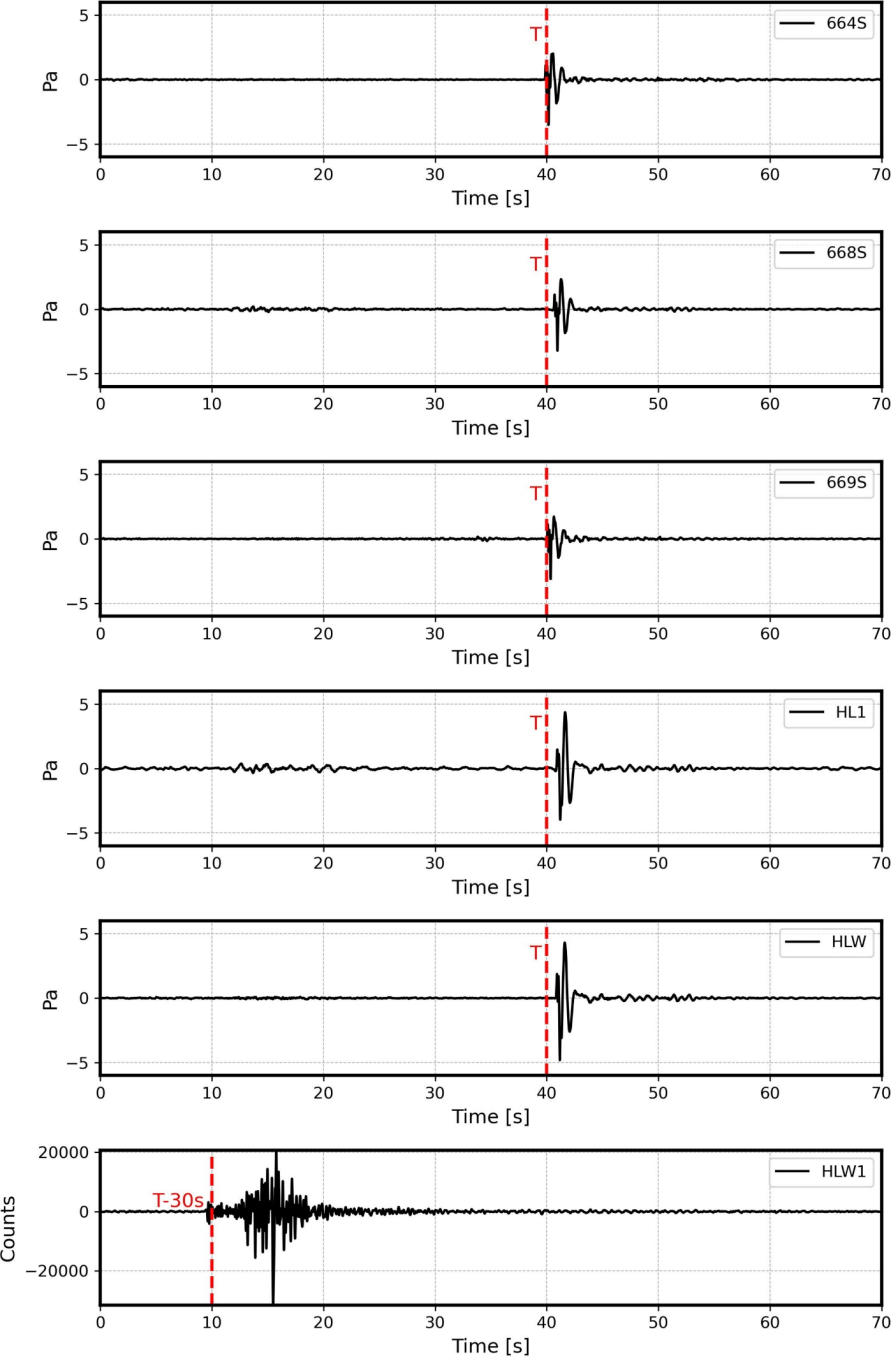
Time delay between seismic wave arrival at HLW1 and acoustic arrivals at 664S, 668S, 669S, HL1, and HLW from the quarry blast at 07:54:51 UTC on 20 November 2022. The signals were filtered between 0.5 and 6 Hz.

The N-shaped waveforms of the detected shockwaves were sharp and clear in most of the events, implying that they were likely recorded from sources close to the Helwan array. Moreover, the time difference between the P-wave and acoustic arrivals from the collocated sensors is approximately 30 s, as illustrated in Fig. 5. Li et al.^30^ analyzed the seismic waveforms produced by quarry blasts to assess their effectiveness in imaging subsurface structures. Additionally, the study estimated the true source location and origin time of these blasts using semblance analysis for P, S, and air waves. By examining different velocity values and computing semblance, the method successfully determined the most probable origin time. Assuming a sound speed of 340 m/s (ignoring the effects of atmospheric conditions) and an average P-wave speed of 6 km/s, the distance between the source and receiver can be estimated using the following formula: $\text {t}_{\text {P-wave}} = \frac{\text {v}_{\text {P-wave}} - \text {v}_{\text {acoustic}}}{\text {v}_{\text {acoustic}}} \times 30$, where $\text {v}_{\text {P-wave}}$ and $\text {v}_{\text {acoustic}}$ represent the velocities of the P-wave and acoustic waves, respectively.

### Array analysis

**Fig. 6:**
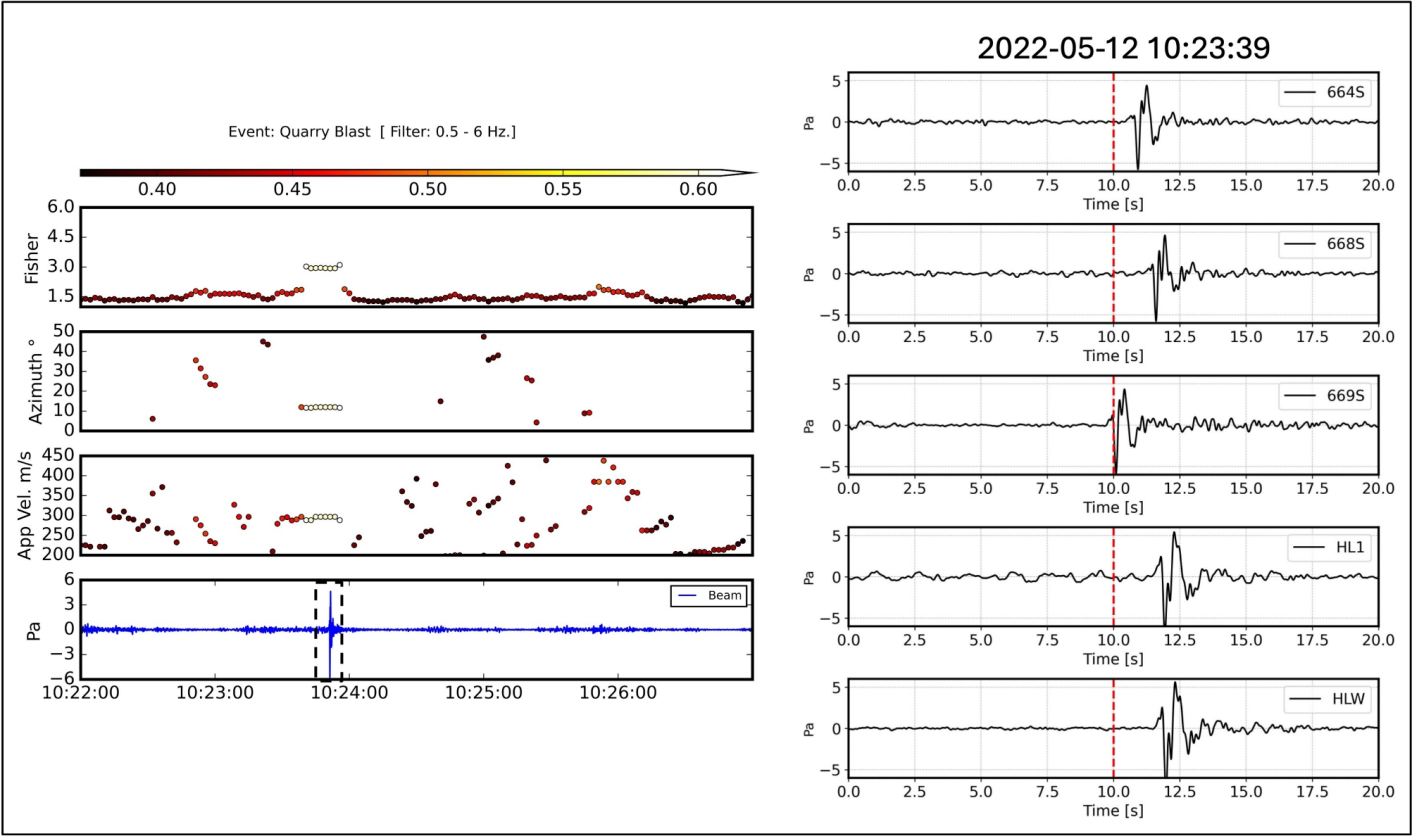
F-K analysis of the event observed on 10:23:49.6 UTC 12 May 2023. Right panel shows a zoomed view of the 20 s surrounding the trigger time, and the signals are filtered between 0.5 and 6 Hz.

F-K analysis was implemented on these 101 filtered events. The window length is 20 s, with 90% overlap. A bandpass filter was applied from 0.5 to 6 Hz. Apparent velocities, back-azimuths, semblance, and the Fisher ratio were estimated from the array analysis for all events. Figure 6 shows an example of F-K analysis applied to an event with a triggered arrival (10:23:49.6 UTC on 12 May 2023).

**Fig. 7:**
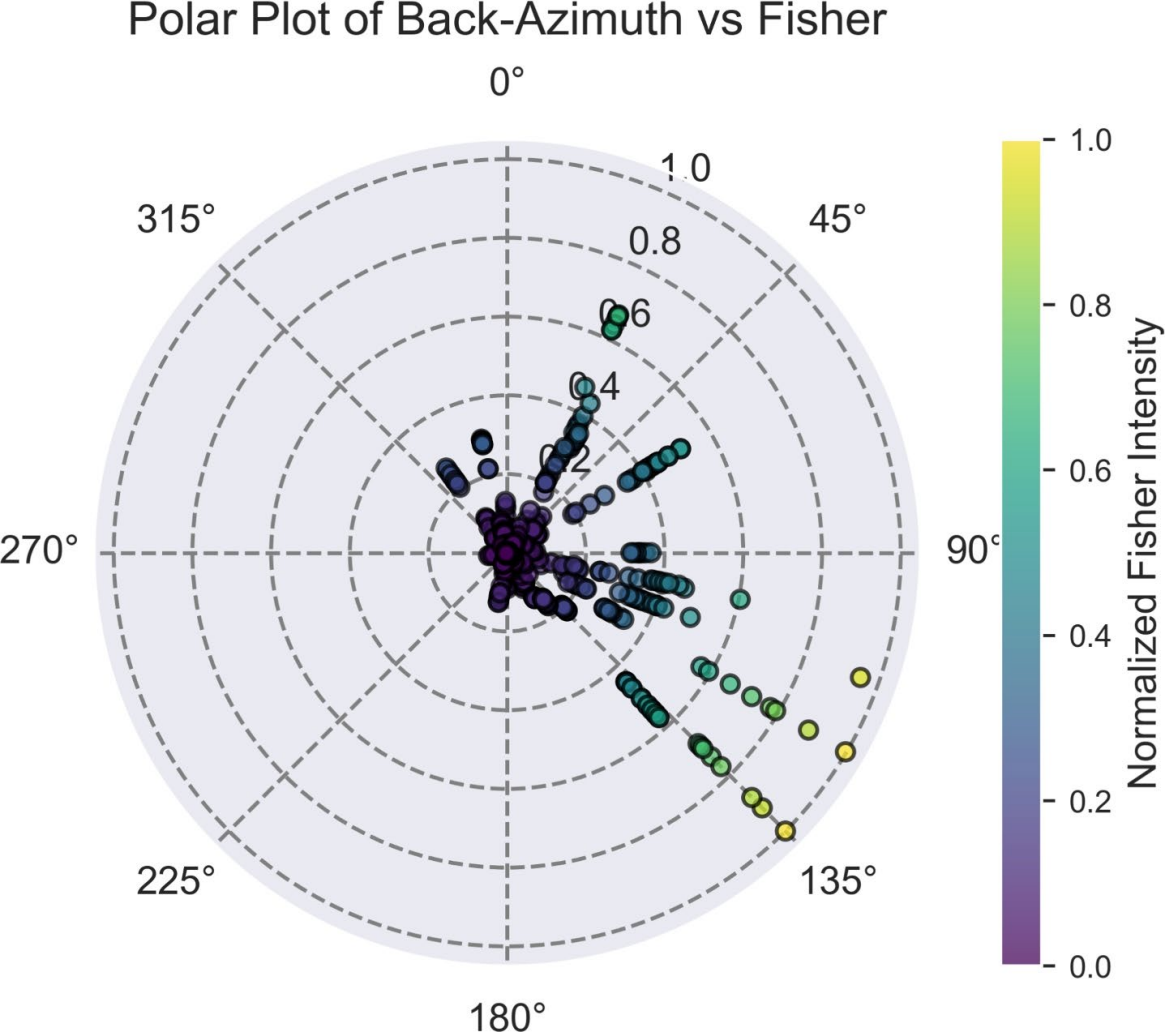
Polar plot of back-azimuth directions with normalised Fisher intensities for all detected infrasound triggers.

The estimated back-azimuths range from 314$^{\circ }$ to 135$^{\circ }$, as shown in Fig. 7. All of the Fisher intensities were normalised between 0 and 1. The majority of detections were from back-azimuths ranging from 120$^{\circ }$ to 135$^{\circ }$ with high normalised Fisher intensities (> 0.4).

**Fig. 8:**
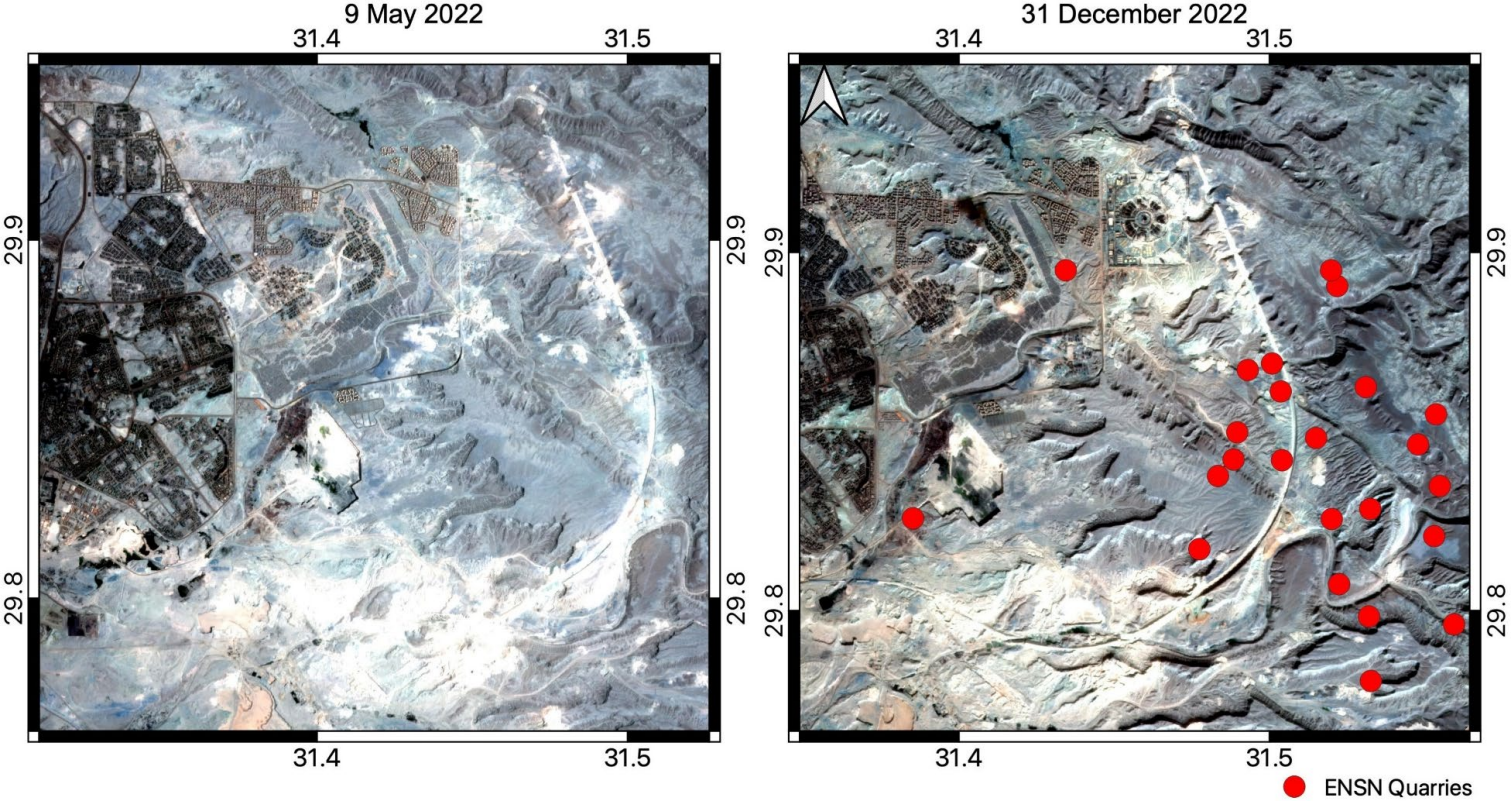
The two panels refer to satellite images captured on 9 May and 31 December 2022. The map was exported using QGIS version 3.8^32^.

The locations of the mysterious events were distributed throughout eastern Helwan, which does not include the zone of mining quarries. The distance between these events and the Helwan array was about 20 km. Two satellite images^31^ were extracted as they represented the suspicious area at the beginning and end of the study period, as shown in Fig. 8. The image from 9 May 2022 shows the initial steps of railway construction and that from 31 December 2022 shows further advancement. This provides visual evidence that the mysterious seismoacoustics could be linked to this project.

### Atmospheric conditions and infrasound propagation models

Atmospheric profiles from the Daily European Centre for Medium-Range Weather Forecasts^33^ were generated over a central point of detected events with latitude 29.8 N and longitude 31.4 E. These profiles are provided up to an altitude of 80 km. PE models were estimated for all daily profiles at a frequency of 4.5 Hz and a propagation distance of 50 km.

For a specific atmospheric profile, let $p_k$ represent the estimated 1D transmission loss at point *k*, where $k=1, 2, 3, ...., n$, and *n* is the total number of transmission loss (tloss) points. The probability distribution functions (PDFs) for the monthly average of the PE models were estimated using the Epanechnikov kernel (K)^34,35^ as shown in Eqs. 9 and 10.

9 $$\begin{aligned} & K(x) = {\left\{ \begin{array}{ll} \frac{3}{4}(1 - x^2) & \text {for } |x| \le 1 \\ 0 & \text {otherwise} \end{array}\right. } \end{aligned}$$

10 $$\begin{aligned} & PDF(p) \approx \frac{1}{nh} \sum _{k=1}^{n} K\left( \frac{p - p_k}{h}\right) , \end{aligned}$$

The bandwidth *h* of the kernel density estimation can be calculated using the following formula:

11 $$\begin{aligned} h = 1.06 \cdot \sigma \cdot n^{-1/5} \end{aligned}$$

where $\sigma$ is the standard deviation of tloss.

**Fig. 9:**
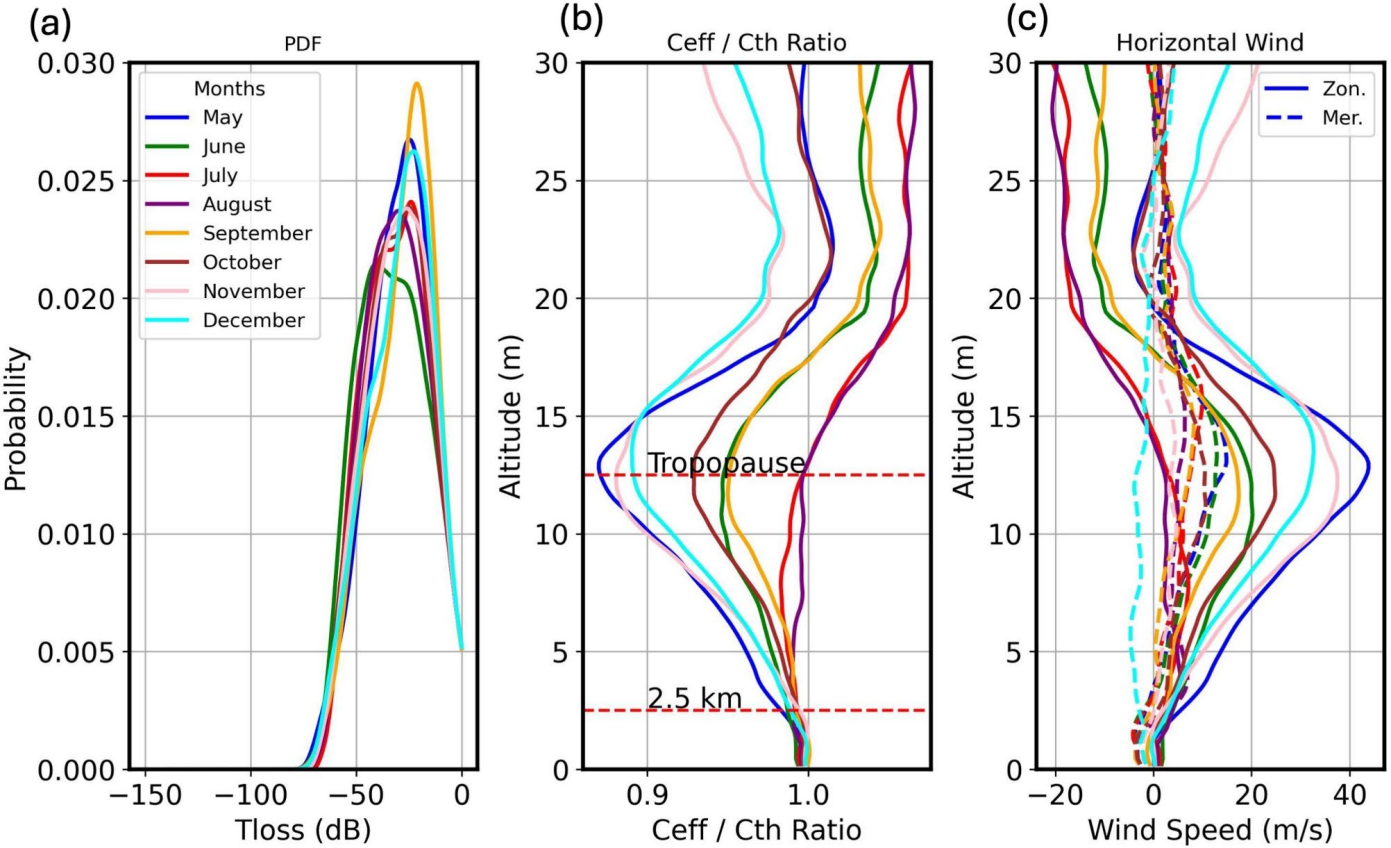
(**a**) Monthly average PDFs derived from the PE model at 4.5 Hz and a propagation distance 50 km. (**b**) Average monthly ratio of $c_{eff}$ and $c_{th}$ toward the Helwan array. (**c**) Average monthly horizontal wind speed for the daily atmospheric profiles over the area of the detected events.

The average zonal wind reached its peak of 45 m/s in May, blowing towards the east at approximately the tropopause altitude. Conversely, its minimum value occurred in November. Strong eastward jets may affect the long-range propagation of infrasound in the westward direction. The effective sound speed ($c_{eff}$) plays a vital role in infrasound propagation, and it can be estimated using $c_{eff}(z)= c_{th}(z)+ \sin ({\phi . u(z)}) +\cos {\phi . v(z)})$, where *u* and *v* are the zonal and meridional wind speeds at latitude *z*, respectively, and $\phi$ is the azimuth direction of propagation, i.e. the wind direction. A comparison between the thermodynamic speed $c_{th}$ and $c_{eff}$ can provide an overview of the propagation conditions in a specific azimuth direction. $c_{th}$ can be derived from: $c_{th}(z)=\sqrt{{\gamma RT}}$ , where $\gamma$ (ratio of specific heat) = 1.4 and *R* (specific gas constant for dry air) = 286.9 J $kg^{-1 }$ $K^{-1}$. The monthly average ratios of $c_{eff}$ and $c_{th}$ indicated that at shorter propagation distances (< 2.5 km), detections could be expected in the direction of azimuth 285$^{\circ }$ towards the Helwan array. Moreover, the monthly average PDFs ranged between 0.02 and 0.03 for losses smaller than -50 dB in all months of the study, as shown in Fig. 9, as all of the events were recorded from a distance of around 20 km. The effect of infrasound propagation in such cases could be considered negligible for these short distances.

## Discussion

Different techniques can be integrated to understand the nature of unknown quarry blasts. Several experiments have been conducted to calibrate the charge of TTE explosions using seismoacoustic signals. Some explosion events can be classified as quarry blasts. While seismometers can detect P-waves as the first signals that arrive from such blasts, they are not a definitive tool for discriminating between natural and artificial events. Additionally, artificial sources generate acoustics that can be captured by infrasound sensors.

Within the Helwan array, different types of infrasound sensor have been tested. The use of inexpensive microphone-type sensors for detecting explosive events could be convenient for local quarries and events with high-frequency signals. However, for lower-frequency signals related to events such as tsunamis, these sensors might not be suitable for recording long-period signals. Moreover, reducing environmental noise at each site is a significant challenge for infrasound array deployment. Noise from both atmospheric conditions and high wind speeds can reduce the probability of infrasonic detections. However, by stacking a large number of inexpensive sensors, the signal-to-noise ratio can be enhanced, potentially offering a convenient solution to reduce noise in specific environments. Furthermore, different noise-reduction systems have been used in the Helwan array to assess the validity of applying such filters. The environmental noise at the site presents a challenge due to the desert environment of Cairo and the traffic around the deployment location.

Applying a trigger method such as the recursive STA/LTA algorithm to the continuous data and comparing the trigger times with the ENSN bulletin enhances the identification of unexplained events. Moreover, in our study, the array analysis of the infrasound sensors corroborated the direction of detected events, linking them to the construction of the high-speed railway. Satellite images also confirmed the progress of the railway construction before and after the study period.

However, the atmospheric conditions during the study period suggested strong ducting towards the east (the opposite side of the Helwan array). Nevertheless, due to the short distances of these events from the array, infrasound propagation models indicated that the decay in pressure amplitudes would be < -50 dB for a frequency of 4.5 Hz. In such cases, the propagation effect might not significantly affect the detections at the Helwan array.

## Conclusions

Since the beginning of 2022, unexplained quarry blasts have been recorded in the ENSN catalogue. The locations of these quarries differed from known mining sites. In this study, several methods were used to investigate these events. The Helwan infrasound array, comprising five infrasound sensors with a collocated seismometer, was deployed at the beginning of May 2022. The setup of the Helwan array elements included different infrasound sensors incorporating a wind-caused noise-reduction system. The use of inexpensive INF04 infrasound sensors demonstrates their value as a cost-effective solution for monitoring higher-frequency events.

The STA/LTA trigger method successfully identified the impulsive signals. Comparing the results against the ENSN quarry catalogue, 85 out of 101 events were recognised. F-K analysis revealed the directions of the quarry blasts. In addition, satellite imagery provided robust evidence of the nature and characteristics of these blasts. Moreover, the monthly average PDFs from infrasound propagation models were used to confirm the locations of the blasts.

Although strong atmospheric jets favoured propagation away from our array, the propagation models confirmed that the blasts arrived from a short distance ($\sim$ 20 km) away.

In conclusion, the integration of different seismoacoustic analysis techniques, along with satellite imagery, allowed for precise discrimination of quarry blasts in eastern Helwan. The unexplained blasts originated from the construction site of a high-speed train railway around Helwan city. Finally, our study shows that quarry blasts related to civil construction can be monitored and tracked using infrasound technology.

## Data Availability

The datasets used and/or analysed during the current study are available from the corresponding author upon reasonable request.
